# Validation of the educational effectiveness of a mobile learning app to improve knowledge about MR image quality optimisation and artefact reduction

**DOI:** 10.1007/s13244-018-0635-0

**Published:** 2018-06-14

**Authors:** Walaa Alsharif, Michaela Davis, Louise Rainford, Andrea Cradock, Allison McGee

**Affiliations:** 1 Radiography and Diagnostic Imaging, School of Medicine, University College Dublin, Dublin, Ireland; 2 Faculty of Applied Medical Sciences, Taibah University, Madinah, Kingdom of Saudi Arabia

**Keywords:** App, MRI, Image quality, Knowledge, Confidence

## Abstract

**Aim:**

The aim was to design an app-based eLearning tool to provide radiographers with information about the physical basis of MR artefacts and practical elimination or/and minimisation strategies to optimise image quality, and to evaluate the impact of a smartphone app on radiographers’ knowledge.

**Methods:**

The study used the comparison-experimental approach (pre- and post-test). Thirty-five MR radiographers independently reviewed a prepared series of MR images (*n* = 25). The participants were requested to identify image quality related errors, to specify error-correction strategies and to score how confident they were in their responses. Participants were then divided into experimental (*n* = 19) and control cohorts (*n* = 16). The app was provided to the experimental cohort for 3 months; after this period both cohorts re-reviewed the MR image datasets and repeated their identification of image quality errors.

**Results:**

The results showed a statistically significant difference between control and experimental cohorts relative to participants’ pre- to post-test knowledge level. For the experimental cohort, years of experience, qualification and type of hospital were not associated with radiographer knowledge level and confidence in recognising the presence of an image quality error, naming the error and specifying appropriate correction strategies (*p* > 0.05).

**Conclusion:**

The study identified the potential of the smartphone app as an effective educational tool to support MR radiographers’ knowledge in recognising and characterising MR image quality errors.

**Key Points:**

• *A high level of knowledge to optimise MR image quality is crucial.*

• *Ongoing education in image quality optimisation is required.*

• *The potential role of app as an effective educational tool is identified.*

## Introduction

Magnetic Resonance Imaging (MRI) is a complex modality based on specific scientific principles and requires a high level of knowledge and expertise for optimal scanning of both routine and complex examinations [1, 2]. Nieboer et al. (2014) stated that healthcare professionals should feel competent to use medical imaging technology in order to provide adequate health care [3]. Literature has endorsed the need for further training and education for radiographers in the area of diagnostic image quality optimisation [4–6]. As a consequence of technology now being an integral part of daily life, this study considered the use of a smart phone application (app) as an opportunity to provide MR radiographers with portable access to learning material. Several researchers have acknowledged that learning opportunities can arise in many formats including web blogs, lectures and eLearning [7–9]. Smartphones have provided healthcare professionals with the opportunity to download apps from a online stores to suit their professional needs. Flannigan and McAloon (2011) and Shaw et al. (2012) refer to the recent trend of using apps in medicine, including diagnostic imaging [10, 11]. Payne et al. (2012) and Lane et al. (2011) highlighted an increasing level of app usage among medical students and doctors [8, 9]. Similar results were found by Alfahad (2009), Dasari et al. (2011) and Ozdalga et al., (2012), Alturki (2013) and more recently by Koh et al. (2014) [12–16].

Several studies have included the testing of knowledge following use of mobile learning [17–19]. Elfeky and Masadeh (2016) and Brize-Ponce (2016) confirmed that the use of mobile learning (e.g., apps) was more effective on students’ knowledge than the use of traditional teaching approaches, due to the availability of the device without the restrictions of time and place [17, 18]. This concurred with the findings of Ling et al. (2014) and Bidaki et al., (2013) who outlined the positive impact of mobile learning apps on academic performance because of the combination of portability and accessibility [19, 20]. While reports such as these highlight the positive utility of educational app, negative effects or caveats related the use of smart phone apps have been reported by various authors. Jung (2012) and Lepp et al. (2015) highlighted the potential distractions from learning due to the ability to connect to social media (e.g., Facebook, Snapchat, and Instagram) [21, 22]. Lewis et al. (2014) also referred to some issues associated with the use of smart phone applications in relation to cost and security issues. Although most the above studies focused on the impact of app usage on students’ learning, the principle of using an app to source information and gain knowledge can be applied to any other group of people such as healthcare professionals, including MR radiographers [23].

Fralick et al. (2017) and Kim et al. (2017) highlighted the positive change in medical staff knowledge following use of an assigned app compared to those who did not have access to this app, as the app promoted their interest in using this technology to learn and the ability to access information repeatedly [24, 25]. To the researcher’s knowledge, there is no study in the literature which focuses on the impact of apps on radiographers’ knowledge. The researcher proposes to test a specifically designed app addressing topics relating to MRI which practitioners will access via their mobile phones to determine the impact on radiographers’ knowledge as they become engaged with the app.

In phase one of a study published in a recent article, a pre-test MR image-based experiment was conducted on a cohort of MR radiographers working in the Kingdom of Saudi Arabia (KSA), without providing them with any learning resources, in order to investigate their level of knowledge regarding MR image-quality-related errors and to identify any potential gaps in their knowledge [4]. In a subsequent phase of this study, reported here, the researcher aimed to:

- Design an app-based eLearning tool provide radiographers with information about the physical basis of MR artefacts and practical elimination or/and minimisation strategies to optimise image quality.
- Test the effect on the knowledge level of the same cohort of MR radiographers relative to MR image quality characteristics and optimisation strategies following a defined period of access to the app-based educational information.

## Methods

### Ethical approval

Ethical approval was attained from the relevant Institutional Review Board (IRB) and directors of the participating diagnostic imaging departments in Saudi Arabia.

### The design of the eLearning tool (app)

**Fig. 1:**
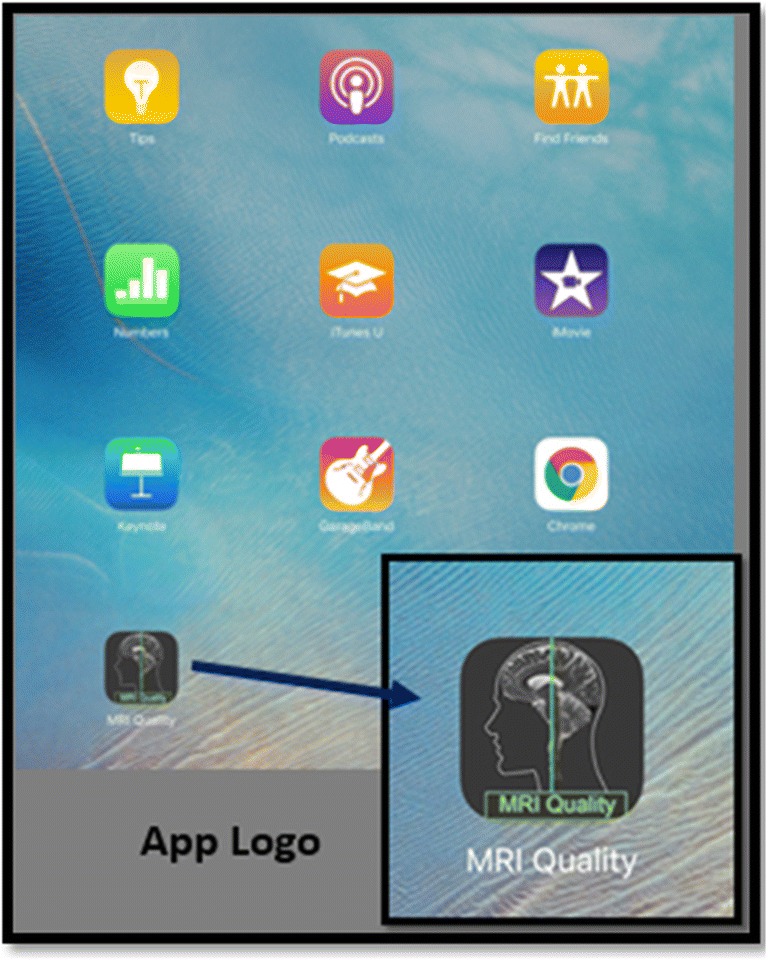
A screen shot of the app logo

**Fig. 2:**
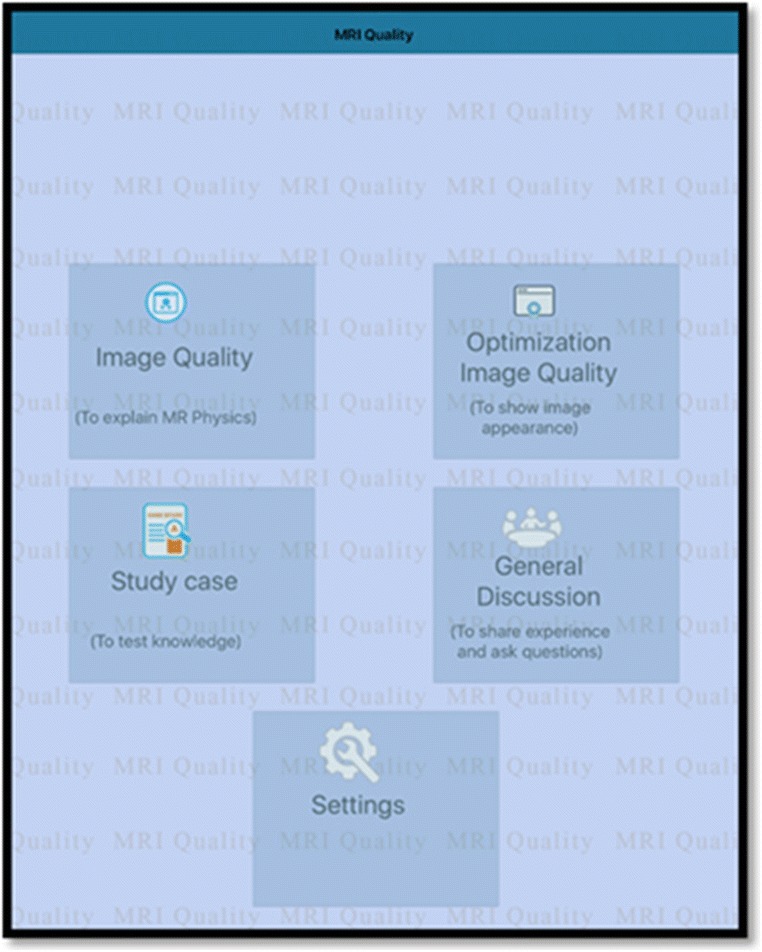
A screen shot of the app contents page

**Fig. 3:**
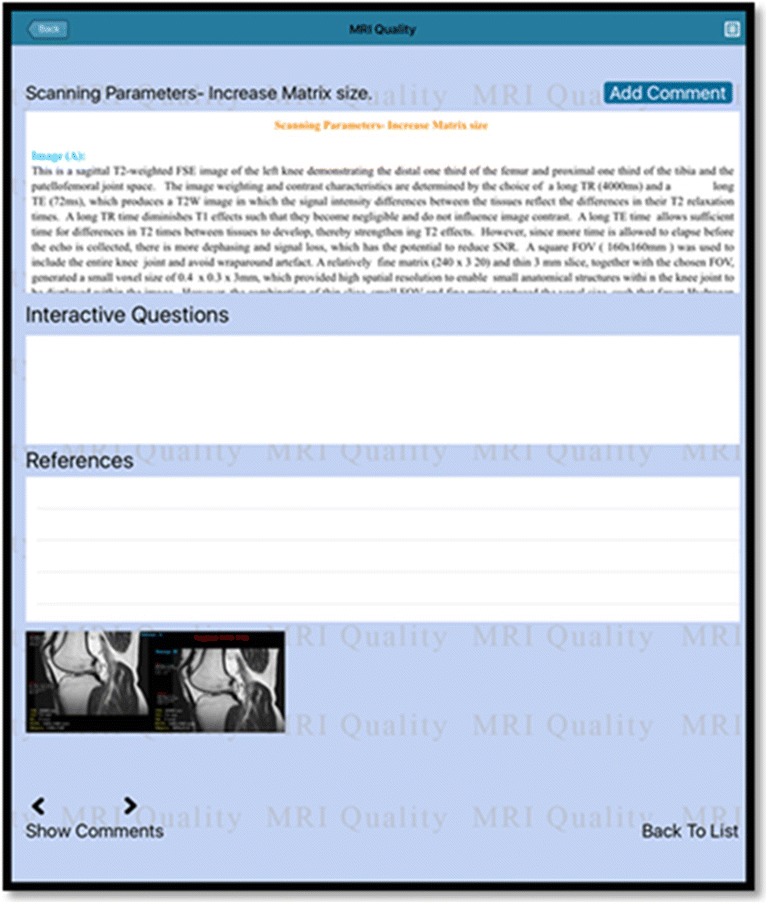
A screen shot showing a sample of the app content

The app content included background information relating to aspects of MR physics that influence MR image quality and associated MR image appearances, together with strategies for optimising image quality. In addition, a platform was included to support an image-based quiz to enable users to test their knowledge, together with one to facilitate app users in the sharing of their clinical expertise regarding MR image quality issues, as shown in Figs. 1, 2 and 3. The app content was verified by a panel of experts comprising one clinical specialist MR radiographer and two lecturers with both clinical and academic expertise in MRI. The researcher then made modifications to the explanatory content and supporting MR images based on feedback received from the panel members. A pilot study was conducted before embarking on the main study, which involved independent review of app functionality by three academic lecturers to assess the design layout of the material presented in the app, determine the practical utility of the various functions of the app, and propose and changes to improve the look and functionality of the app. Modifications were made to the app based on the comments received during the pilot phase.

### Image-based experiment

In order to evaluate the impact of the app on the level of the MR radiographers’ knowledge, group one (*n* = 19) (experimental group) was given access to the app for a three-month period, and group two (*n* = 16) acted as the control group and had no access to this app. Radiographers were randomly selected and allocated to group one or two so that both groups comprised radiographers with similar levels of academic qualification and number of years of clinical MR scanning experience working in similar hospital types. The sample size was calculated to give sufficient power (at least 80%) for comparing the proportion of MR images correctly identified as demonstrating the presence or absence of an image-quality error between groups. A one-tail proportion test was used to calculate the sample size for the post-test groups (experimental and control), assuming alpha = .05 to detect 10% difference in answers from each radiographer group. The researchers used their own professional backgrounds supported by the literature to identify MR image quality errors that radiographers may typically encounter in their clinical practice. The MR image datasets (*n* = 25) were selected from among those acquired from clinical MR scanners in the KSA to represent a range of brain, spine, musculoskeletal, abdominal and pelvic MR images typically acquired in the clinical setting, which represented the features of excellent, acceptable and poor image quality. A pilot study was conducted with MR clinical specialists (*n* = 2) and an academic lecturer to validate the MR image datasets and the questions to be asked of participating radiographers. The viewing condition and time of image viewing session (40 min) were similar to the earlier pre-test phase of this study as previously reported [4]. Upon completion of the initial image viewing session (pre-test) [4], the experimental group was provided with access to the app. Literature recommends a short intervention duration (>6 months) to facilitate the engagement with the task activity and maintain the enthusiasm of participants in using app technology [26–28]. In line with this, a three-month period was allocated for app usage, after which time the image-based experiment (post-test) was undertaken. MR radiographers in both the experimental and control groups independently reviewed the MR image dataset, and were required to answer the questions outlined in Table 1.

**Table 1:** Questions used in the image experiment to evaluate radiographers’ knowledge with regard to MR image-quality errors

Questions	
A- Indicate if an artefact and/or other technical error is present within an MR image B- Identify this/ these artefacts’/ technical errors C- Outline how this/ these artefacts’/ technical errors can be corrected, reduced or eliminated D- Indicate the degree of confidence in your responses by using scale 0-100 - in naming the type of MR image quality error; - in specifying the error-correction strategy for the MR image quality error	

### Statistical analysis

Statistical analysis was performed using IBM SPSS version 20, with a *p* value of <0.05 considered significant. Descriptive analysis, Wilcoxon signed-rank test and multiple linear regression were used for data analysis.

## Results

MR radiographers (*n* = 35) working in hospitals (*n* = 16) public and semi-public (military, academic) in Saudi Arabia participated (Table 2). The MR images (*n* = 25) reviewed by participants included images with several image quality errors as shown in Table 3.

**Table 2:** Demographic profile of the participants

Group		Hospital Type			Years of Experience		Qualification	
		Public	Military	Academic	1-5	6+	Diploma	Degree
Pre-test		13	13	9	21	14	8	27
Post-test	Experimental	6	6	4	9	7	4	12
	Control	7	7	5	12	7	4	15

**Table 3:** The percentage of images within the MR image dataset

Image	Frequency	Percentage
SNR (Signal to noise ratio)	4	16%
CNR (Contrast to noise ratio)	6	24%
SR (Spatial resolution)	2	8%
Artefact	7	28%
SNR and CNR	1	4%
No quality errors evident	5	20%
Total	25	100%

Table 4 demonstrates the change in radiographers’ responses from pre- to post-test in recognising the existence of the image quality errors for the experimental and the control groups. The radiographers’ ability to recognise the existence of the image-quality errors was significantly different in the experimental group who had access to the app (Z = 3.54, *p* < 0.001).

**Table 4:** Variations in radiographer responses to the recognition of MR image-quality errors

Error Category	Group	Number of images	Total Number of responses	Number (%) correct (pre-test)	Number (%) Dis-improved	Number (%) incorrect (pre-test)	Number (%) improved
Image without errors	Control Group	5	80	61 (76.3%)	8 (12.9%)	19 (23.8%)	8 (42.1%)
	Experimental Group		95	86 (90%)	9 (12.3%)	22 (23.2%)	19 (86.4%)
Image with errors	Control Group	20	320	314 (98.1%)	6 (2.1%)	39 (12.2%)	4 (10.3%)
	Experimental Group		380	375 (98.7%)	5 (1.5%)	37 (9.7%)	34 (91.9%)

The change of MR radiographers’ responses from pre- to post-test in identifying the type of the image quality errors by the type of error is shown in Table 5 for both groups. The ability of radiographers to correctly name the type of image quality error present was significantly different in the experimental group (Z = 3.73, *p* < 0.001).

**Table 5:** Variations in radiographer responses in identifying different types of MR image-quality errors

Error Category	Group Control N = 16 Experimental N = 19	Number of images	Total Number of responses	Number (%) correct (pre-test)	Number (%) Dis-improved	Number (%) incorrect (pre-test)	Number (%) improved
SNR (Signal to noise ratio)	Control	4	64	40 (62.5%)	7 (16.3%)	24 (37.5%)	6 (25%)
	Experimental		76	44 (57.9%)	15 (34.1%)	32 (42.1%)	18 (56.3%)
CNR (Contrast to noise ratio)	Control	6	96	67 (69.8%)	10 (15.9%)	29 (30.2%)	7 (24.1%)
	Experimental		114	65 (57.02%)	0 (0%)	49 (43%)	40 (81.6%)
SR (Spatial resolution)	Control	2	32	11 (34.4%)	3 (27.2%)	21 (65.6%)	4 (19%)
	Experimental		38	17 (44.7%)	0 (0%)	21 (55.3%)	21 (100%)
Artefact	Control	7	112	51 (45.5%)	4 (7.8%)	61 (54.5%)	4 (6.6%)
	Experimental		133	74 (55.6%)	3 (4.1%)	59 (44.4%)	37 (62.7%)
No Technical Error Evident	Control	5	80	62 (77.5%)	7 (11.3%)	18 (22.5%)	6 (33.3%)
	Experimental		95	73 (76.9%)	7 (9.6%)	22 (23.2%)	19 (86.4%)
SNR and CNR	Control	1	16	10 (62.5%)	2 (20%)	6 (37.5%)	0 (0%)
	Experimental		19	15 (78.9%)	0 (0%)	4 (21.1%)	4 (100%)

Table 6 shows the change in MR radiographers’ responses from pre- to post-test in specifying the appropriate error-correction strategy for the image quality errors for both participant groups. A significant difference was found in the radiographers’ ability to specify the appropriate error-correction strategy in the experimental group (Z = 3.83, *p* < 0.001).

**Table 6:** Variation in radiographer responses in specifying the appropriate error-correction strategy for MR image-quality errors

Correction Strategy	Group Control N = 16 Experimental N = 19	Number of images	Total Number of responses	Number (%) correct (pre-test)	Number (%) Dis-improved	Number (%) incorrect (pre-test)	Number (%) improved
Change Sequence parameters	Control	4	64	15 (23.4%)	4 (26.7%)	49 (76.6%)	5 (10.2%)
	Experimental		76	17 (22.4%)	1 (5.9%)	59 (77.6%)	42 (71.2%)
Patient setup issue	Control	2	32	19 (59.4%)	5 (26.3%)	13 (40.6%)	1 (7.7%)
	Experimental		38	28 (73.7%)	0 (0%)	10 (26.3%)	8 (80%)
Change timing parameters	Control	3	48	19 (39.6%)	4 (21.1%)	29 (60.4%)	2 (6.9%)
	Experimental		57	22 (38.6%)	0 (0%)	35 (61.4%)	34 (97.1%)
Artefact	Control	3	48	24 (50%)	3 (12.5%)	24 (50%)	2 (8.3%)
	Experimental		57	27 (47.4%)	1 (3.7%)	30 (52.6%)	19 (63.3%)
Correct RF coil	Control	3	48	12 (25%)	4 (33.3%)	36 (75%)	5 (13.9%)
	Experimental		57	18 (31.6%)	6 (33.3%)	39 (68.4%)	16 (41%)
Change scanning technique	Control	5	80	19 (23.8%)	5 (26.3%)	61 (76.3%)	3 (4.9%)
	Experimental		95	23 (24.2%)	3 (13%)	72 (75.8%)	56 (77.8%)
No technical error evident	Control	5	80	62 (77.5%)	6 (9.7%)	18 (22.5%)	6 (33.3%)
	Experimental		95	73 (76.9%)	7 (9.6%)	22 (23.2%)	19 (86.4%)

A reduction in radiographers’ confidence level in identifying the image quality error and/or error-correction strategy was noted in the experimental group from pre- to post-test compared to the control group. The results show no change in median of change in confidence level for the both groups (Table 7).

**Table 7:** A table demonstrating the confidence level in identifying the type of the image quality errors and specifying an appropriate error-correction strategy for both groups

			Pre-test confidence level	Post-test confidence level	Change in confidence level
Confidence level in identifying the type of the image quality errors	Group	Valid N	Median (1RQ)	Median (IRQ)	Median (IRQ)
	Control	16	90 (80-100)	90 (80-100)	0 (0-0)
	Experimental	19	90 (80-100)	85 (80-85)	0 (−5-0)
Confidence level in specifying the error-correction strategy	Control	16	80 (72-95)	80 (80-98)	0 (0-0)
	Experimental	19	85 (75-90)	80 (80-85)	0 (−5-5)

Multiple linear regression of radiographers’ change in knowledge demonstrated a significant difference in radiographers’ ability to correctly recognise the existence of image quality errors, name the type of the quality errors and specify the error-correction strategy between participants in the experimental and control groups (*p* < 0.001) (Table 8).

**Table 8:** A table demonstrating a multiple linear regression used to evaluate the impact of the app on radiographers’ knowledge

	Model	Unstandardised Coefficients		Standardised Coefficients	t	*p*-value	95.0% Confidence Interval for B		R square
		B	Std. Error	Beta			Lower Bound	Upper Bound	
Recognise the existence of the image quality errors	Intercept	11.502	2.782		4.134	<0.001	5.835	17.169	0.57
	Access to the app	2.460	0.443	0.651	5.556	<0.001	1.558	3.362	
	Pre-test	−.544	0.129	−.493	−4.207	<0.001	−.807	−.281	
Identifying the type of the image quality errors	Intercept	8.892	1.709		5.202	<0.001	5.410	12.374	0.783
	Access to the app	6.434	0.700	0.757	9.191	<0.001	5.008	7.860	
	Pre-test	−.615	0.108	−.469	−5.686	<0.001	−.836	−.395	
Specifying the appropriate correction strategy	Intercept	6.539	1.935		3.380	<0.002	2.599	10.480	0.802
	Access to the app	9.912	0.905	0.862	10.948	<0.001	8.068	11.756	
	Pre-test	−.657	0.171	−.302	−3.841	<0.001	−1.005	−.308	

In all three regression models, the coefficients were negative (B = −.544, −.615, −.657) for the number of correct responses in the pre-test, confirming that those who displayed a high level of knowledge in the pre-test had less improvement from pre- to post-test. Interactions of use of the app with years of clinical experience, qualification and type of employing hospital were investigated, but none were found to be significant (Table 8).

A multiple linear regression demonstrated that no significant difference was found in median change in radiographer confidence level in naming the type of image quality errors and specifying the error-correction strategy between those who had access to the app and the control group (*p* = 0.609 and 0.700, respectively).

**Fig. 4:**
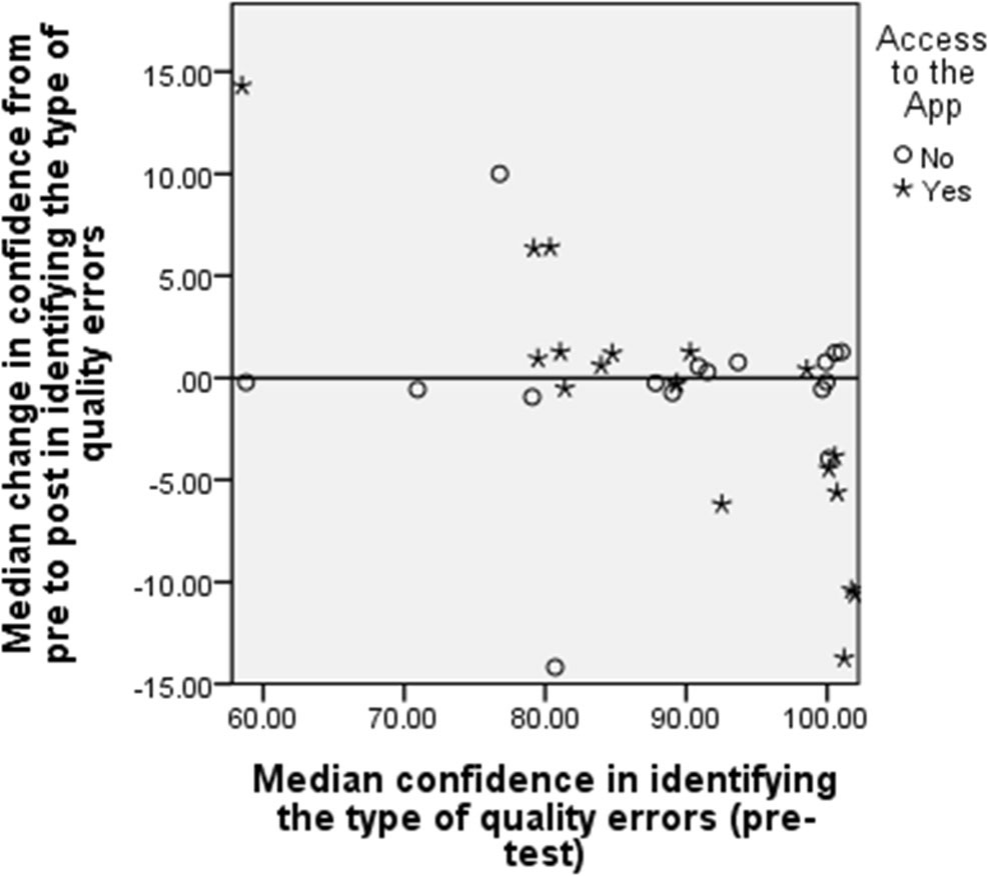
Scatterplot of median change in confidence level for radiographers in identifying the type of image-quality errors verse pre-test confidence by experimental group

The results show that the overall change in median confidence level in naming the type of image-quality errors tended to be close to zero, with little difference between those in the experimental and control groups. There was more variability in median change in the confidence level for the experimental group than for the control group. A number of radiographers in the experimental group had a very high confidence level in the pre-test, with a median decrease in confidence from pre- to post-test (Fig. 4).

**Fig. 5:**
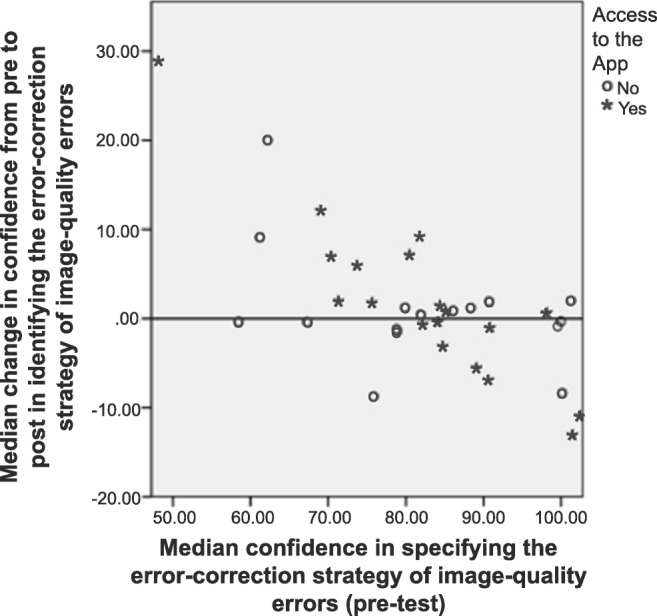
Scatterplot of median change in confidence level for radiographers in specifying the error-correction strategy of image-quality errors verse pre-test confidence by experimental group

Figure 5 shows that the median change in confidence level in specifying the error-correction strategy tended to be higher for those who had low median change in confidence level in pre-test, and lower for those who had high median change in confidence level in pre-test. This was expected, as radiographers with high confidence have less capacity to improve. There appears to be little difference in median change in confidence level in specifying the error-correction strategy between experimental and control groups.

In both regression models for confidence level, the coefficients were negative for pre-test confidence levels, confirming that those who showed a high level of confidence in the pre-test had less increase in level of confidence from pre- to post-test (Table 9). Interactions between use of the app with years of clinical experience, qualification and type of hospitals were investigated and none were found to be significant.

**Table 9:** A table demonstrating a multiple linear regression used to evaluate the impact of the app on radiographers’ level of confidence in reviewing MR image quality

	Model	Unstandardised Coefficients		Standardised Coefficients	t	p-value	95.0% Confidence Interval for B		R square
		B	Std. Error	Beta			Lower Bound	Upper Bound	
Confidence level in identifying the type of the image quality errors	Intercept	22.267	3.603		3.295	<0.002	8.502	36.032	0.231
	Access to the App	−.876	1.696	−.078	−.516	0.609	−4.331	2.579	
	Pre-test	−.258	0.075	−.519	−3.447	<.002	0.410	−.106	
Confidence level in specifying the appropriate correct strategy	Intercept	35.856	6.587		5.443	<0.001	224.38	49.274	0.454
	Access to the App	0.790	2.035	0.049	0.378	0.700	−3.355	3.238	
	Pre-test	−.770	0.078	−.696	−5.492	<0.001	−.590	−.271	

## Discussion

A smartphone-based educational app was used in this study to highlight commonly encountered MR image quality issues. The app was provided to one group of MR radiographers (experimental group) for a three-month period, after which the image-based experiment was repeated in order to evaluate the impact of the app on MR radiographers’ ability to evaluate MR image quality.

Several studies in the literature have been conducted to evaluate the effectiveness of mobile learning apps on knowledge [17–20]. Bidaki et al., (2013) and Brize-ponce et al., (2016) found a positive impact of apps on student knowledge due to the flexibility and accessibility offered, and student interest to learn via apps [18, 19]. This was similarly highlighted by Elfeky and Masadeh (2016) and Ling et al., (2014) [17, 20]. In the current study, the results revealed that those who had access to the app showed an improvement in their ability to correctly appraise MR image quality. This was in line with Fralick et al., (2017) and Kim et al., (2017) who referred to the positive change in medical staff knowledge in the experimental group compared to the control group from pre- to post-test [24, 25]. The findings in the current study demonstrate a high improvement in responses from pre- to post-test for the experimental group when the quality errors were related to SR (100%), CNR (81.6), SNR combined with CNR (100%). However, dis-improvement in radiographer performance was evident when the error was related to the SNR (15%). The potential reasons for this low level of radiographer performance may have been due to a negative mood, or a heavy workload on the day of the experiment as highlighted by Caruso (2014) and Cavallo et al., (2002) [29, 30]. Individual radiographer’s attitudes toward using the app may have influenced their perceptions about the usefulness of the app. Some factors that could have influenced an individual radiographer’s attitude include time, interest, engagement, boredom and technical ability [31, 32]. Even though a radiographer might have a positive attitude toward using the app, they might still have ended up not using the app on a consistent basis due to a lack of time, work commitments and limited access to the internet. However, the potential effect of these factors was not explored in the current study.

Further findings show no improvement in responses for the control group from pre- to post-test when the quality error was related to the SNR, or SR combined with CNR. Although participants in the control group did not have access to the app, participants appeared to have improved in their knowledge of some of the topics relating to MR image quality such as SR (19%) and SNR (25%). This slight improvement in responses of the participants in the control group from pre- to post-tests may be attributed to events which took place before the post-test (e.g., short courses, in-house training), that may have influenced radiographers’ ability to correctly recognise and categorise image quality errors in the post-test phase. Radiographers may also have gained knowledge related to image quality through independent reading of journal articles or books. Participants may have reviewed some material/ topics regarding image-quality errors, or they may have tried to retrieve images from their memory in response to their awareness of the pre-test experiment.

Additionally, the improvement in responses from pre- to post-test was marked for both groups in specifying the appropriate error-correction strategies when these were related to patient setup (e.g., patient immobilisation, patient instructions), change of timing parameters and change of scanning technique (e.g., BLADE, 3D sequence). Although both groups showed a level of improvement from pre- to post-test in similar topics, this improvement in responses was lower in the control group compared to the experimental group. In contrast, dis-improvement in responses was evident between both groups when the correction strategies were related to appropriate RF coil selection. A possible reason for this is that radiographers in the experimental group did not have sufficient time to review this topic through the app, or this topic may require a further explanation in the app. Since radiographers in this phase of the study were not asked for their feedback and satisfaction level regarding the app content, this limits the conclusion that can be made.

It appears that radiographers in the experimental group were able to correctly answer more questions relating to image quality errors compared to the control group (*p* < 0.001). This may reflect the positive impact of the app on radiographers’ knowledge. However, it is important to note that some MR radiographers may have had opportunities to access a training course prior to the post-test phase, and this may have impacted their responses. Additionally, the pre-test phase may also have had an impact, as participation in the pre-test image review phase may have increased their awareness about image quality and may have stimulated them to do some independent reading and learning on this topic. Other factors that could have impacted on radiographers’ knowledge are the memory effect and long-term retention. Relative to other studies aimed to test healthcare staff knowledge at different time intervals [24, 25, 33, 34], the total time interval used in this study was 1 year to facilitate app design, and to minimise the possibility of participating radiographers recalling image appearances from the pre-test phase. It is important to acknowledge that time intervals required between pre- and post-tests are varied and depend upon the study objectives and aims [35]. Although the gap between the pre- and post-tests was 1 year, radiographers may have remembered the image appearances from the pre-test phase, and their memory may have influenced their responses in the post-test phase.

Further, results indicate that those who showed a high level of knowledge and confidence in the pre-test phase showed less improvement from pre-test to post-test. This is not surprising, as radiographers who showed high levels of knowledge and confidence in the pre-test phase had less capacity to show high improvement in post-test; they can show only no change or dis-improvement in their responses and level of confidence. In the current study, use of the app with years of clinical experience, qualification and type of hospitals were not associated with the radiographers’ level of knowledge and confidence in recognising the existence of image quality errors, naming the error and specifying an appropriate correction strategy for each image. This would appear to indicate that the app was equally effective for radiographers who worked in different hospital types, with different years of clinical experience and level of academic qualification.

The findings highlight a slight drop in confidence level for those in the experimental group compared to the control group in the post-test phase. The potential reason for this may be the feeling of being tested, which in turn may have impacted the radiographers’ inherent confidence levels in their responses. For example, radiographers may have initially felt confident, but this may have translated to under-confidence as they perceived their knowledge was being tested. In addition to, access to the app and the retest experiment may have created uncertainty in radiographers’ responses. For example, radiographers may have thought that they knew enough about a particular topic, but on accessing the app, they felt they were not sure about their knowledge. This finding is contrary to the recent studies undertaken by Ling et al., (2014) and Kim et al., (2017) who refer to higher scores on confidence in students’ performance and knowledge following engagement with prescribed learning materials [20, 25].

The study findings have demonstrated an improvement in radiographer responses in identifying the quality errors and specifying the appropriate error-correction strategy for the group who had access to the app compared to those who had no access. The findings tend to indicate the potential of smartphone apps as an effective educational tool to bridge the gap in MR radiographers’ knowledge in reviewing image quality, as smartphones are very convenient compared to textbooks due to ease of use, portability and availability as online components. This was in line with the findings of Kim et al., (2017) who indicated smartphone apps are an effective method for use in nursing education [25]. However, it is important to note that a period of time between pre-test and designing the app, and the post-test could have impacted on changes in radiographers’ responses. This one-year time period may have provided the opportunity for other developments to occur besides receiving access to the app. For example, in the interim, the radiographers may have had an opportunity to access learning opportunities such as short courses or articles. However, long time intervals between the pre- and post-tests could help to minimise participant recall of information from the pre-test phase [36, 37]. The researchers in the current study did not ask the participants if they had access to other learning materials during the time interval between the pre- and post-test phases, but there is a possibility that radiographers could have done so, which potentially may have impacted the results. Additionally, since a similar image experiment was conducted in the pre- and post-tests, radiographers might have shown an improvement in their responses simply due to the fact that they had seen the test images before.

## Limitations

Although, the sample size was statistically sufficient, it was relatively small for checking whether true associations exist between use of the app with years of clinical experience, qualification and type of hospitals and further research with a larger sample size is warranted. The memory effects and long-term retention when the individual MR radiographers were retested is uncertain. It is also acknowledged that this study was confined to the MR radiographers who work in public, military and academic hospitals in the three main regions in Saudi Arabia. Therefore, the results may not be confidently generalised to the MR radiographers who work in private hospitals in the KSA, or in other regions across the country.

## Conclusion

The study findings show an improvement in radiographer responses from pre- to post-test in recognising the existence of the image-quality errors, naming the errors and specifying the error-correction strategy for the experimental group compared to the control group from pre- to post-test. The app was equally effective for radiographers who worked in different hospital types, with different clinical experience and level of academic qualification. The study encourages the use of smartphone apps as an accessible and effective educational tool to bridge the gap in MR radiographers’ knowledge in reviewing image quality.
